# Role of puboperinealis and rectourethralis muscles as a urethral support system to maintain urinary continence after robot-assisted radical prostatectomy

**DOI:** 10.1038/s41598-023-41083-8

**Published:** 2023-08-29

**Authors:** Masao Kataoka, Satoru Meguro, Ryo Tanji, Akifumi Onagi, Kanako Matsuoka, Ruriko Honda-Takinami, Seiji Hoshi, Junya Hata, Yuichi Sato, Hidenori Akaihata, Soichiro Ogawa, Motohide Uemura, Yoshiyuki Kojima

**Affiliations:** Departments of Urology, Fukushima Medical University School of Medicine, 1 Hikarigaoka, Fukushima, 960-1295 Japan

**Keywords:** Oncology, Urology

## Abstract

The present study investigated the role of a urethral support system to maintain urinary continence after robot-assisted radical prostatectomy (RARP), with a focus on pelvic floor muscles, such as the puboperinealis muscle (PPM) and rectourethralis muscle (RUM). Finally, 323 patients who underwent RARP were analyzed in this study. All patients performed a one-hour pad test 1, 3, 6, 9, and 12 months after RARP to assess urinary incontinence and MRI before and 9 months after RARP to evaluate the pelvic anatomical structure. The preoperative cross-sectional area of PPM (2.21 ± 0.69 cm^2^) was significantly reduced by 19% after RARP (1.79 ± 0.60 cm^2^; p < 0.01). Positive correlations were observed between the amount of urinary leakage according to the 1-h pad test 1, 3, 6, 9, and 12 months after RARP and the change in the cross-sectional area of PPM by RARP (p < 0.01, < 0.001, < 0.001, < 0.001, and < 0.001, respectively). A positive correlation was also noted between the amount of urinary leakage 6 and 12 months after RARP and the preoperative RUM diameter (p < 0.05). The amount of urinary leakage 1, 3, 6, 9, and 12 months after RARP negatively correlated with the change in the antero-posterior diameter of the membranous urethra (MU diameter) from the static to dynamic phases during the Valsalva maneuver by cine MRI. Furthermore, the change in the MU diameter negatively correlated with the change in the cross-sectional area of PPM (p < 0.05). PPM and RUM play significant roles as a supportive mechanism to maintain urinary continence by functioning as a urethral support.

## Introduction

Although robot-assisted radical prostatectomy (RARP) has recently become the gold standard treatment for localized prostate cancer, postoperative urinary incontinence is still a complication that decreases the quality of life (QOL) of patients^1^. Preoperative risk factors for urinary incontinence after prostatectomy include an advanced age, higher BMI, larger prostate volume, severe preoperative lower urinary tract symptoms, and a history of prostatectomy for benign prostatic hyperplasia^2^. Preoperative anatomical variables measured on magnetic resonance imaging (MRI), such as the length of the membranous urethra (MU) and periurethral supporting structures, have also been associated with urinary incontinence after RARP^3^. However, the mechanisms underlying the development of urinary incontinence have not yet been examined in detail.

The levator ani muscle is an important anatomical and functional structure for maintaining urinary continence, and its preservation improves urinary continence^4–7^. It comprises the puborectalis, pubococcygeus, and iliococcygeus muscles. The puboperinialis muscle (PPM) is the medial part of the puborectalis, arising from the posterior surface of the pubis on each side and passing posteriorly beside the urethra^8^. The rectourethralis muscle (RUM) is located at the interface between PPM and the rectum. Right and left PPM muscle edges are located close together in an area between the urethra and rectum, immediately lateral to RUM. Since these structures may form a strong sling and influence the stabilization of the MU, we considered the preoperative status of and surgical damage to these structure to potentially be important factors contributing to urinary incontinence after RARP. In the present study, we conducted an anatomical analysis of the structures around the urethra using MRI before and after RARP, and discussed the mechanisms underlying postoperative urinary incontinence.

## Methods

### Patients

We conducted a prospective observational cohort study. Patients who underwent RARP in our hospital between 2014 and 2019 were included in the present study. Patients with organ-confined prostate cancer were considered to be candidates for RARP and were included in the study. Inclusion criteria were (1) clinically localized prostate cancer, (2) age < 75 years, and (3) an Eastern Cooperative Oncology Group performance status of 0. During this period, 533 patients who met the inclusion criteria underwent RARP. The Ethics Committee of Fukushima Medical University Hospital approved this study (approval number: 2614) and all patients provided written informed consent. This study was performed in accordance with the ethical standards laid down in the 1964 Declaration of Helsinki.

### Surgical technique

RARP was performed using a four-arm da Vinci Si™ surgical system (Intuitive Surgical Inc., Sunnyvale, CA, USA) by the transperitoneal posterior approach reported by Guillonneau et al. in conventional laparoscopic procedures^9^. This approach is initiated with transverse peritoneotomy between the bladder and rectum, followed by retrovesical dissection. After dissection of the bladder neck, the prostatic vascular pedicles are ligated and the prostate is removed. Preservation of the neurovascular bundles is recommended to patients with low or favorable intermediate risk prostate cancer. Posterior reconstruction was performed on all cases in the present study. Vesicourethral anastomosis was conducted using the Van Velthoven procedure. RARP was performed or supervised by a single surgeon (Y.K.).

### One-hour pad test to evaluate urinary incontinence

Urinary incontinence was evaluated by the one-hour pad test recommended by the International Continence Society^10^. We considered it important to perform the assessment of urinary incontinence objectively because the anatomical analysis was objectively conducted by MRI. Patients performed the one-hour pad test 1, 3, 6, 9, and 12 months after RARP to evaluate the amount of urinary leakage. Pad test information was collected by urology nurses.

### MRI examinations and imaging techniques

**Figure 1:**
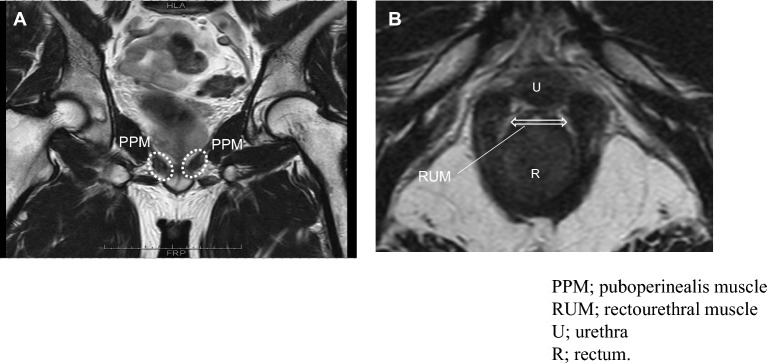
Anatomical analysis of the structure around the urethra using MRI before and after RARP. (**A**) The left and right cross-sectional areas of PPM (dotted circles) at the height of RUM on the dorsal side of the urethra were calculated using coronal sections. (**B**) The tissue between the urethra and rectum was considered to be RUM (double arrow) in the transverse plane, and the left–right diameter was measured. *PPM* puboperinealis muscle, *RUM* rectourethral muscle, *U* urethra, *R* rectum.

MRI was performed before and 9 months after RARP in all cases because the effects of postoperative inflammation in muscles needed to be excluded. Static and dynamic MRI were performed using a 1.5 T MR scanner (Magnetom Trio; Siemens Healthcare, Erlangen, Germany). Dynamic MRI consisted of a T2-weighted, single slice True Fast Imaging with Steady-state Procession (TrueFISP) sequence in the sagittal, coronal, and transverse planes. The cross-sectional area of PPM and left–right (transverse) diameter of RUM (RUM diameter) were measured on T2-weighted fast spin echo (FSE) sequences. The left and right cross-sectional areas of PPM at the height of RUM on the dorsal side of the urethra were calculated using coronal sections, and the sum of the left and right cross-sectional areas was defined as the cross-sectional area of PPM (Fig. 1A). The tissue between the urethra and rectum was considered to be RUM in the transverse plane, and the left–right diameter was measured (Fig. 1B).

**Figure 2:**
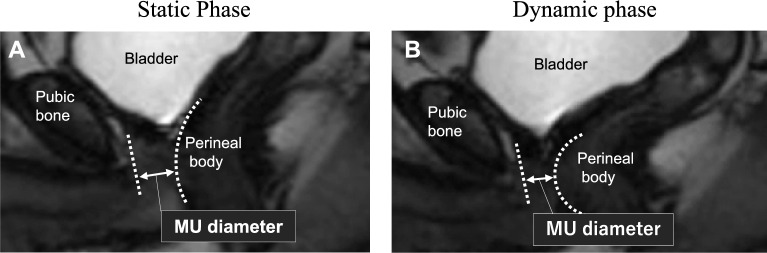
Antero-posterior diameter of the membranous urethra (MU diameter) in static (**A**) and dynamic phases (**B**) during the Valsalva maneuver by cine MRI.

Patients also underwent cine MRI to evaluate the antero-posterior MU diameter with a full bladder 9 months after RARP (Fig. 2). Cine MRI in static and dynamic phases was conducted during the Valsalva maneuver and T2-weighed sagittal sections were obtained. Cine acquisition was performed over a 30-s time period during the static and dynamic phases. A urologist provided patients with guidance in the examination room during MRI scanning. Changes in the MU diameter from the static (Fig. 2A) to dynamic phases (Fig. 2B) during the Valsalva maneuver by cine MRI were calculated.

### Statistical analysis

All data were analyzed using SPSS statistical package version 24.0 (SPSS Inc., Chicago, IL, USA). The significance of differences was considered when p < 0.05. Parametric comparisons between before and after RARP were performed using the paired *t*-test. Simple and multiple regression models were used to examine the relationship between two or more variables. Variables with p < 0.05 in the simple regression model were included in the multiple regression model.

## Results

**Figure 3:**
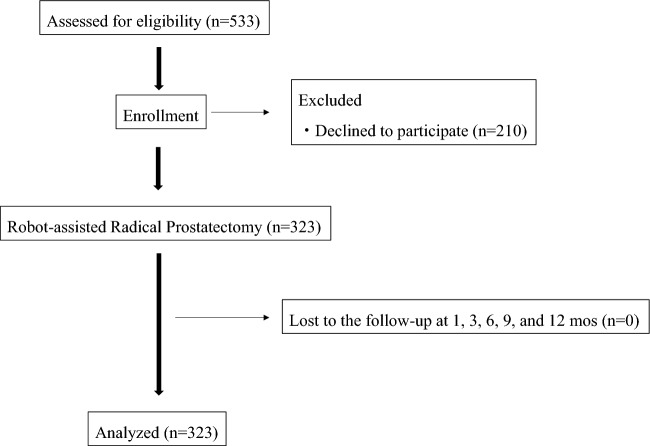
Flowchart of the study.

Finally, 323 patients (mean age, 67.2 ± 6.0 years; age range, 48–76 years) were enrolled in the present study (Fig. 3). The baseline and perioperative characteristics of patients are shown in Table 1. The average amounts of urinary leakage in the one-hour pad test obtained by weighing the pads were 5.4, 46,1, 22.1, 18.5, 14.3, and 13.0 ml before and 1, 3, 6, 9, and 12 months after RARP, respectively. When urinary continence was defined as total urine leakage of < 2 g in the one-hour pad test^10^, continence rates were 85.1, 44.6, 75.5, 75.5, 76.5, and 78.0% before and 1, 3, 6, 9, and 12 months after RARP, respectively (Table 2).

**Table 1:** Patient characteristics.

Parameters	Data
Preoperative physical status	
Patient age (years)	67 (49–77)
Body height (cm)	165.0 (147.5–181.9)
Body weight (kg)	67.6 (48.0–111.9)
BMI (kg/m^2^)	24.5 (17.1–33.5)
Preoperative anti-androgen (no.)	
Yes	46
No	277
Disease characteristic	
Prostate size (cm^3^)	45.3 (20.0–110.0)
Initial serum PSA (ng/mL)	10.4 (3.70–44.2)
Clinical stage (no.)	
T1	
T1c	86
T2	
T2a	146
T2b	27
T2c	42
T3	
T3a	19
T3b	3
Pathological stage (no.)	
T2	
T2a	79
T2b	25
T2c	123
T3	
T3a	73
T3b	23
N1	
T3aN1	1
T3bN1	3
Surgical events	
Operation time (min)	269 (125–494)
NVB preservation (no.)	
No	249
Unilateral	64
Bilateral	10

**Table 2:** Amount of urinary leakage according to the 1-h pad test and the rate of urinary continence before and 1, 3, 6, 9, and 12 months after RARP.

	Before RARP	After RARP				
		1 M	3 M	6 M	9 M	12 M
Volume of urinary leakage (ml)	5.4 (0–184)	46.1 (0–447)	22.1 (0–287)	18.5 (0–316)	14.3 (0–289)	13.0 (0–276)
Rate of urinary continence (%)	85.1	44.6	75.5	75.5	76.5	78.0

We compared the cross-sectional area of PPM and RUM diameter using MRI before and after RARP (Table 3). The preoperative cross-sectional area of PPM (2.21 ± 0.69 cm^2^) was significantly reduced by 19% after RARP (1.79 ± 0.60 cm^2^; p < 0.01). No significant change was observed in the RUM diameter between before and after RARP (1.33 ± 0.28 and 1.36 ± 0.29 cm, respectively). There was no significant correlation between the change in the cross-sectional area of PPM and preoperative anti-androgen administration in the simple regression model (p = 0.49). There was also no significant correlation between the change in the cross-sectional area of PPM and neurovascular bundle (NVB) preservation in the simple regression model (p = 0.17). In addition, this correlation was not found regardless of unilateral or bilateral neurovascular bundle (NVB) preservation (p = 0.26 and 0.13, respectively).

**Table 3:** Comparison of the PPM cross-sectional area and left–right diameter of RUM before and after RARP.

	Pre-RARP	Post-RARP	p-value
PPM (cross-sectional area; cm^2^)	2.21 ± 0.69	1.79 ± 0.60	< 0.01
RUM (left–right diameter; cm)	1.33 ± 0.28	1.36 ± 0.29	n.s

*PPM* puboperinealis muscle, *RUM* rectourethral muscle, *RARP* robot-assisted radical prostatectomy.

We performed a correlation analysis between the amount of urinary leakage according to the one-hour pad test after RARP and BMI, neurovascular bundle (NVB) preservation during RARP or prostate volume, which are considered to be factors which may affect urinary continence^11^. In the simple regression model, the amount of urinary leakage 1 or 3 months after RARP positively correlated with BMI (p < 0.01 and < 0.05, respectively). The amount of urinary leakage 1, 6, or 9 months after RARP negatively correlated with NVB preservation during RARP (p < 0.05, respectively). The amount of urinary leakage 6 months after RARP positively correlated with prostate volume (p < 0.05; Table 4). The multiple regression model revealed a correlation between the amount of urinary leakage 1 months after RARP and BMI or NVB preservation (p < 0.01 and p = 0.05, respectively; Table 5).

**Table 4:** Comparison between the amount of urinary leakage by the 1-h pad test and the PPM cross-sectional area or left–right diameter of RUM before and after RARP: simple regression analysis.

After RARP	1 month		3 months		6 months		9 months		12 months	
	β (95% CI)	p-value	β (95% CI)	p-value	β (95% CI)	p-value	β (95% CI)	p-value	β (95% CI)	p-value
BMI	0.15 (0.01–0.04)	< 0.01	0.11 (0.00–0.03)	< 0.05	0.08 (− 0.00–0.03)	0.16	0.03 (− 0.01–0.02)	0.64	0.11 (− 0.00–0.03)	0.06
NVB preservation	− 0.12 (− 0.27 to – 0.02)	< 0.05	− 0.07 (− 0.21–0.03)	0.17	− 0.12 (− 0.24 to – 0.00)	< 0.05	− 0.12 (− 0.23 to – 0.01)	< 0.05	− 0.09 (− 2.01–0.02)	0.11
Prostate volume	0.05 (− 0.00–0.01)	0.36	0.06 (− 0.00–0.01)	0.30	0.12 (0.00–0.01)	< 0.05	0.07 (− 0.00–0.01)	0.20	0.10 (0.00–0.01)	0.07
PPM (cross-sectional area)										
Pre-RARP	0.02 (− 0.61–0.10)	0.45	0.05 (− 0.72–0.08)	0.89	0.03 (− 0.05–0.10)	0.46	− 0.01 (− 0.08–0.06)	0.70	0.02 (− 0.05–0.10)	0.53
Post-RARP	− 0.11 (− 0.20 to − 0.02)	< 0.05	− 0.19 (− 0.27 to − 0.10)	< 0.001	− 0.18 (− 0.26 to − 0.10)	< 0.001	− 0.17 (− 0.25 to − 0.10)	< 0.001	− 0.17 (− 0.24 to − 0.09)	< 0.001
Changes before and after RARP	0.20 (0.10–0.31)	< 0.001	0.30 (0.20–0.40)	< 0.001	0.33 (0.24–0.42)	< 0.001	0.24 (0.15–0.34)	< 0.001	0.30 (0.21–0.39)	< 0.001
RUM (left–right diameter)										
Pre-RARP	0.19 (− 0.01–0.38)	0.06	0.27 (0.08–0.46)	< 0.01	0.30 (0.13–0.48)	< 0.01	0.20 (0.03–0.37)	< 0.05	0.34 (0.17–0.50)	< 0.001
Post-RARP	0.35 (0.17–0.53)	< 0.001	0.33 (0.15–0.51)	< 0.001	0.34 (0.17–0.50)	< 0.001	0.26 (0.10–0.42)	< 0.01	0.36 (0.20–0.51)	< 0.001
Changes before and after RARP	0.29 (0.06–0.52)	< 0.05	0.14 (− 0.09–0.36)	0.25	0.10 (0.57–4.84)	0.36	0.12 (− 0.08–0.33)	0.24	0.09 (− 0.12–0.29)	0.40

*BMI* body mass index, *NV* neurovascular bundle, *PPM* puboperinealis muscle, *RUM* rectourethral muscle, *RARP* robot-assisted radical prostatectomy.

Change in PPM before and after RARP: preoperative PPM − postoperative PPM.

Change in RUM before and after RARP: preoperative RUM − postoperative RUM.

**Table 5:** Comparison between the amount of urinary leakage by the 1-h pad test and the PPM cross-sectional area or left–right diameter of RUM before and after RARP: multiple regression analysis.

After RARP	1 month		3 months		6 months		9 months		12 months	
	Β (95% CI)	p-value	Β (95% CI)	p-value	Β (95% CI)	p-value	Β (95% CI)	p-value	Β (95% CI)	p-value
BMI	0.15 (0.01–0.04)	< 0.01	0.01 (− 0.00–0.03)	0.08						
NVB preservation	− 0.11 (− 0.25 to – 0.01)	< 0.05			− 0.79 (− 0.19 to – 0.23)	0.12	− 0.08 (− 0.20–0.02)	0.11		
Prostate volume					0.06 (− 0.00–0.01)	0.28				
PPM (cross-sectional area)										
Pre-RARP										
Post-RARP	− 0.05 (− 0.14–0.04)	0.26	− 0.13 (− 0.21 to − 0.42)	< 0.01	− 0.11 (− 0.19 to − 0.04)	0.43	− 0.13 (− 0.20 to − 0.05)	< 0.01	− 0.10 (− 0.47 to − 0.03)	< 0.01
Changes before and after RARP	0.17 (0.06–0.28)	< 0.01	0.26 (0.16–0.36)	< 0.001	0.30 (0.20–0.39)	< 0.001	0.20 (0.11–0.30)	< 0.001	0.27 (0.18–0.36)	< 0.001
RUM (left–right diameter)										
Pre-RARP	0.19 (− 0.01–0.38)	0.06	0.18 (− 0.59–0.42)	0.14	0.24 (0.02–0.45)	< 0.05	0.13 (− 0.09–0.35)	0.25	0.26 (0.05–0.47)	< 0.05
Post-RARP	0.30 (0.09–0.50)	< 0.01	0.14 (− 0.09–0.37)	0.23	0.11 (− 0.01–0.32)	0.30	0.11 (0.10–0.32)	0.31	0.12 (− 0.73–0.32)	0.22
Changes before and after RARP	0.05 (− 0.21–0.30)	0.72								

*BMI* body mass index, *NV* neurovascular bundle, *PPM* puboperinealis muscle, *RUM* rectourethral muscle, *RARP* robot-assisted radical prostatectomy.

Change in PPM before and after RARP: preoperative PPM − postoperative PPM.

Change in RUM before and after RARP: preoperative RUM − postoperative RUM.

We performed a correlation analysis between the amount of urinary leakage according to the one-hour pad test after RARP and MRI parameters, such as the cross-sectional area of PPM or RUM diameter (Table 4). In the simple regression model, the amount of urinary leakage 1, 3, 6, 9, or 12 months after RARP negatively correlated with the postoperative PPM cross-sectional area (p < 0.05, < 0.001, < 0.001, < 0.001, and < 0.001, respectively). The amount of urinary leakage 1, 3, 6, 9, or 12 months after RARP positively correlated with the change in the cross-sectional area of PPM by RARP (p < 0.001) and the postoperative RUM diameter (p < 0.001, < 0.001, < 0.001, < 0.01, and < 0.001, respectively). Furthermore, positive correlations were observed between the amount of urinary leakage 3, 6, 9, or 12 months after RARP and the preoperative RUM diameter (p < 0.01, < 0.01, < 0.05, and < 0.001, respectively) as well as between the amount of urinary leakage 1 month after RARP and the change in the RUM diameter by RARP (p < 0.05).

**Figure 4:**
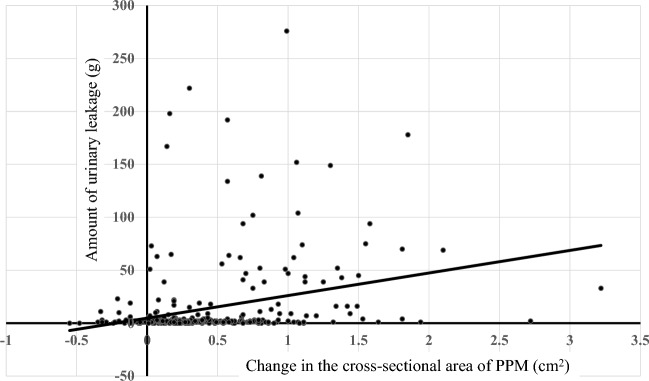
Distribution chart to show the correlation between the change in the cross-sectional area of PPM (before and 12 months after RARP) and amount of urine leakage. Change in PPM before and 12 months after RARP: preoperative PPM − postoperative PPM.

The multiple regression model revealed a negative correlation between the amount of urinary leakage 3, 9, or 12 months after RARP and the postoperative cross-sectional area of PPM (p < 0.01; Table 5). The amount of urinary leakage 1, 3, 6, 9, or 12 months after RARP positively correlated with the change in the cross-sectional area of PPM by RARP (p < 0.01, < 0.001, < 0.001, < 0.001, and < 0.001, respectively). These results indicate that a decrease in the cross-sectional area of PPM by RARP induced urinary incontinence after RARP. Representative distribution chart to show the correlation between the amount of urine leakage 12 months after RARP and the change in the cross-sectional area of PPM is shown in Fig. 4. A positive correlation was noted between the amount of urinary leakage 6 or 12 months after RARP and the preoperative RUM diameter (p < 0.05). Furthermore, the amount of urinary leakage 1 month after RARP positively correlated with the postoperative RUM diameter (p < 0.01). These results indicate that a large RUM diameter, which reflects a large distance between the left and right PPM, is a risk factor for the development of urinary incontinence after RARP.

A correlation analysis of the amount of urinary leakage according to the one-hour pad test after RARP and the change in the MU diameter from the static to dynamic phases during the Valsalva maneuver was also performed (Table 6). The simple regression model revealed a negative correlation between the amount of urinary leakage 1, 3, 6, 9, or 12 months after RARP and the change in the MU diameter (p < 0.001). The multiple regression model also showed that the amount of urinary leakage 1, 3, 6, 9, or 12 months after RARP negatively correlated with the change in the MU diameter (p < 0.001). Although the amount of urinary leakage 1, 3, 6, 9 or 12 months after RARP negatively correlated with preoperative membranous urethral length (p < 0.05, p < 0.05, p < 0.05, p < 0.05 and p < 0.01, respectively) in the simple model, this correlation did not find in the multiple regression model (p = 0.78, p = 0.27, p = 0.33, p = 0.07 and p = 0.30, respectively). It did not correlate with postoperative membranous urethral length in the simple regression model, (p = 0.11, 0.13, 0.33, 0.30 and 0.30, respectively).

**Table 6:** Correlation between the amount of urinary leakage by the 1-h pad test and pre- or post-operative membranous urethral length or the change in MU diameter on cine MRI parameters after RARP.

After RARP	1 month		3 months		6 months		9 months		12 months	
	β (95% CI)	p-value	β (95% CI)	p-value	β (95% CI)	p-value	β (95% CI)	p-value	β (95% CI)	p-value
Simple regression analysis										
Preoperative MUL	− 0.27 (− 0.43 to − 0.12)	< 0.05	− 0.27 (− 0.42 to − 0.11)	< 0.05	− 0.19 (− 0.33 to − 0.04)	< 0.05	− 0.16 (− 0.30 to − 0.02)	< 0.05	− 0.19 (− 0.32 to − 0.05)	< 0.01
Postoperative MUL	0.14 (− 0.06–0.25)	0.11	0.13 (− 0.14–0.30)	0.13	0.09 (− 0.07–0.28)	0.33	− 0.02 (− 0.14–0.03)	0.30	0.10 (− 0.02–0.32)	0.30
Change in MU diameter	− 98.9 (− 132.6 to − 65.4)	< 0.001	− 81.1(− 100.5 to − 61.7)	< 0.001	-69.7 (− 90.9 to − 48.5)	< 0.001	− 48.0 (− 66.3 to − 29.6)	< 0.001	− 46.2 (− 63.5 to − 28.9)	< 0.001
Multiple regression analysis										
Preoperative MUL	0.03 (− 0.17–0.23)	0.78	0.10 (− 0.08–0.29)	0.27	0.09 (− 0.09–0.25)	0.33	0.16 (− 0.01–0.33)	0.07	0.09 (− 0.08–0.25)	0.30
Postoperative MUL										
Change in MU diameter	− 98.7 (− 142.2 to − 55.2)	< 0.001	− 70.6 (− 95.1 to − 46.1)	< 0.001	− 60.2 (− 86.9 to − 33.4)	< 0.001	− 40.2 (− 63.5 to − 16.9)	< 0.001	− 45.3 (− 67.5 to − 23.2)	< 0.001

*PPM* puboperinealis muscle, *RUM* rectourethral muscle, *MU* membranous urethra, *MUL* membranous urethral length, *RARP* robot-assisted radical prostatectomy.

Change in MU diameter: Changes in the MU diameter from the static to dynamic phases during the Valsalva maneuver by cine MRI.

We also examined the relationship between the change in the MU diameter from the static to dynamic phases during the Valsalva maneuver and the cross-sectional area of PPM or the RUM diameter (Table 7). The simple regression model showed that the change in the MU diameter from the static to dynamic phases negatively correlated with the change in the cross-sectional area of PPM (p < 0.01) and the preoperative (p < 0.05) or postoperative RUM diameter (p < 0.05). The multiple regression model revealed a negative correlation between the change in the MU diameter and the change in the cross-sectional area of PPM (p < 0.05).

**Table 7:** Correlation between the change in MU diameter on cine MRI at 9 months after RARP and the PPM cross-sectional area and left–right diameter of RUM before and after RARP.

	Simple regression analysis		Multiple regression analysis	
	β (95% CI)	p-value	β (95% CI)	p-value
PPM (cross-sectional area)				
Pre-RARP	− 0.053 (− 0.054–0.019)	0.34		
Post-RARP	0.064 (− 0.017–0.064)	0.25		
Changes before and after RARP	− 0.152 (− 0.119 to − 0.020)	< 0.01	− 0.151 (− 0.119 to − 0.019)	< 0.05
RUM (left–right diameter)				
Pre-RARP	− 0.113 (− 0.179 to − 0.003)	< 0.05	− 0.088 (− 0.189 to − 0.047)	0.237
Post-RARP	− 0.115 (− 0.172 to − 0.005)	< 0.05	− 0.045 (− 0.146–0.078)	0.548
Changes before and after RARP	0.010 (− 0.096–0.115)	0.85		

*PPM* puboperinealis muscle, *RUM* rectourethral muscle, *MU* membranous urethra.

Change in PPM before and after RARP: preoperative PPM − postoperative PPM.

Change in RUM before and after RARP: preoperative RUM − postoperative RUM.

Change in MU diameter: Changes in the MU diameter from the static to dynamic phases during the Valsalva maneuver by cine MRI.

## Discussion

The present study investigated the relationship between the anatomical pelvic structure around the urethra by MRI and urinary incontinence after RARP, and suggested that a decrease in the postoperative cross-sectional area of PPM after RARP and a large RUM diameter affected the postoperative urethral support system and consequently induced urinary incontinence.

**Figure 5:**
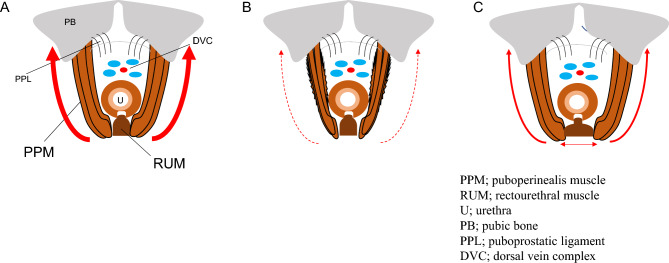
Anatomical structure around the urethra before and after RARP and the mechanism underlying postoperative urinary incontinence. (**A**) PPM and RUM function as a urethral sling to maintain urinary continence. (**B**) Surgical interventions during RARP damage and may lead to the atrophy of PPM and the loss of its function as a urethral sling. (**C**) The longer preoperative distance between left and right PPM indicate looser function as a urethral sling, and these patients may originally have had weak urethral support. *PPM* puboperinealis muscle, *RUM* rectourethral muscle, *U* urethra, *PB*; pubic body, *PPL* puboprostatic ligament, *DVC* dorsal vein complex.

The levator ani muscle plays a significant role in maintaining urinary continence and its preservation improves urinary continence^4–7^. Retzius-sparing RARP, which preserves the levator ani and puboprostatic ligaments, has a higher continence rate than standard RARP^12–15^. PPM, which has components of the levator ani muscle, is responsible for the quick stop phenomenon of urination in males, and, thus, the weakening of PPM by transection, traction injury, or denervation may affect urinary continence after radical prostatectomy^16^. In other words, preserving the integrity and innervation of PPM is crucial for the maintenance of urinary continence^16^. The right and left PPM course posteriorly from the pubis to flank the urethra, and converge in the midline behind the urethra, anterior to the rectum^16^. RUM occupies a space between the right and left PPM^17^. The left and right PPM edges are consistently located in close proximity in an area immediately lateral to RUM^17^, suggesting that PPM and RUM function as a urethral sling to maintain urinary continence^17,18^ (Fig. 5A). This urethral support mechanism by the connection between PPM and RUM is important for urinary continence in women^19,20^. In women, urethral hypermobility and a low urethral closure pressure due to damage to the urethral support mechanism causes stress urinary incontinence^21^. Mid-urethral sling surgery has become the gold standard treatment for stress urinary incontinence in women^22^, and the effectiveness of sling surgery depends on adequate postoperative urethral mobility and the urethral closure pressure^23^. The loss of the prostate may cause instability in the urethral support mechanism in men after RARP, as in women^11^. Therefore, we considered the urethral support mechanism by the connection between PPM and RUM to be important for urinary continence in men after RARP, and that the low ability or weak strength of inherent PPM and RUM and surgical damage to PPM and RUM after RARP may induce urinary incontinence after RARP.

We herein focused on morphological changes in PPM and RUM before and after RARP. Sohn et al. examined the anatomy of men preoperatively with MRI, and found that men with a thicker puborectalis muscle at the anorectal angle regained continence earlier after prostatectomy^24^. We demonstrated that the cross-sectional area of PPM decreased by 19% after RARP. Moreover, the decrease in the cross-sectional area of PPM after RARP correlated with postoperative urinary incontinence and induced inadequate urethral compression. These results suggest that surgical interventions during RARP may damage PPM itself and nerve and/or blood supply around PPM, leading to the atrophy of PPM, the loss of its function as a urethral sling (Fig. 5B), and, as a result, urinary incontinence after RARP. Therefore, to avoid postoperative urinary incontinence by loss of PPM muscle volume, it may be important to minimize damage to nerves and blood supply by refraining from using electrocautery around PPM and minimize mechanical damage to PPM itself from surgical manipulation during RARP. However, NVB preservation may not be able to avoid loss of PPM muscle volume.

RUM also may play a significant role as a urethral support system by its connection with left and right PPM to maintain urinary continence. Individual variations have been reported in the development of RUM^17,18^. Soga et al. showed that RUM influenced MU stabilization with the rhabdosphincter, and also that it was one of the important structures forming a urethral sphincteric complex^18^. In the present study, pre- and postoperative RUM diameters positively correlated with urinary incontinence; however, the change in the MU diameter from the static to dynamic phases during the Valsalva maneuver did not correlate with the pre- or postoperative RUM diameter. Therefore, although the longer pre- and postoperative distances between left and right PPM were not directly proven to indicate weaker pre- and postoperative function as a urethral sling in the present study, these patients may originally have had weak urethral support from before RARP, which may easily induce postoperative urinary incontinence (Fig. 5C). Therefore, the preoperative measurement of RUM by MRI may predict urinary incontinence after RARP.

Preoperative pelvic floor muscle training (PFMT) enhances the postoperative function of pelvic muscles, including the levator ani muscle, reduces urinary incontinence after prostatectomy, and improves QOL outcomes related to urinary continence^25–27^. PFMT with a strength training protocol to hypertrophy the levator ani muscle may yield higher continence rates^25^. PFMT recuperates strength and lost tone in the sling mechanism after prostatectomy, and potentially enhances the pelvic floor support of vesicourethral anastomosis and the bladder base by strengthening PPM^16^. Additionally, the development of surgical procedures to reinforce the urethral sling system may promote urinary continence after RARP. Vladimir et al. reported that the advanced reconstruction of the vesicourethral support technique using the levator ani muscle attenuated urinary incontinence after RARP^28^. Previous studies demonstrated the effectiveness of urethral sling or bladder neck suspension techniques during RARP for urinary continence^29–31^. We showed that bladder neck sling suspension during RARP promoted the early return of urinary continence^29^. Cestari et al. found that a proper sling procedure during RARP allowed for the restoration of the sphincteric apparatus capability to its presurgical status; however, its capability decreased after prostate removal and subsequent ureterovesical anastomosis^30^. The efficacy of the male sling to treat post-prostatectomy incontinence has been reported^32^. Our anatomical data on the relationship between pelvic muscles and urinary incontinence after RARP appear to support the effectiveness of PFMT, surgical procedures, such as the urethral sling or bladder neck suspension techniques, and postoperative male sling procedures to maintain urinary continence after RARP.

There are several limitations that need to be addressed. First, this was a single-center analysis. Furthermore, postoperative MRI was only performed 9 months after RARP in all cases because the effects of postoperative inflammation in muscles need to be excluded. Second, we conducted pre- and postoperative anatomical analyses of the structure around the urethra using MRI, but not a functional analysis, such as the urethral pressure profile. Third, Valsalva effort is variable and subjective. The degree of dynamic movement is influenced by this effort. Therefore, further detailed studies are warranted.

## Conclusions

PPM and RUM play significant roles in the supportive mechanism to maintain urinary continence as a urethral support. Surgical interventions during RARP lead to the atrophy of PPM, the loss of its function as a urethral sling, and, ultimately, urinary incontinence after RARP. The preoperative measurement of PPM by MRI may predict urinary incontinence after RARP. The further development of surgical procedures to prevent or minimize damage to and reinforce this urethral support system may contribute to the mitigation of urinary incontinence after RARP.

## Data Availability

The datasets generated during and/or analysed during the current study are available from the corresponding author on reasonable request.

## Abbreviations

RARP: Robot-assisted radical prostatectomy
PPM: Puboperinealis muscle
RUM: Rectourethral muscle
UI: Urinary incontinence
MU: Membranous urethra

## References

[1] Litwin MS (1995). Quality-of-life outcomes in men treated for localized prostate cancer. JAMA.

[2] Qin H (2019). Predictors for immediate recovery of continence following Retzius-sparing robot-assisted radical prostatectomy: A case–control study. Int. Urol. Nephrol..

[3] Mungovan SF (2017). Preoperative membranous urethral length measurement and continence recovery following radical prostatectomy: A systematic review and meta-analysis. Eur. Urol..

[4] Laucirica O (2020). Complete puborectalis, puboperinealis muscle and urethral rhabdomyosphincter preservation in laparoscopic radical prostatectomy: Anatomical landmarks to achieve early urinary continence. Int. J. Urol..

[5] Song C (2007). Relationship between the integrity of the pelvic floor muscles and early recovery of continence after radical prostatectomy. J. Urol..

[6] Neumann PB, O'Callaghan M (2018). The role of preoperative puborectal muscle function assessed by transperineal ultrasound in urinary continence outcomes at 3, 6, and 12 months after robotic-assisted radical prostatectomy. Int. Neurourol. J..

[7] Muñoz-Calahorro C (2021). Anatomical predictors of long-term urinary incontinence after robot-assisted laparoscopic prostatectomy: A systematic review. Neurourol. Urodyn..

[8] Walz J (2016). A critical analysis of the current knowledge of surgical anatomy of the prostate related to optimisation of cancer control and preservation of continence and erection in candidates for radical prostatectomy: An update. Eur. Urol..

[9] Guillonneau B, Vallancien G (2000). Laparoscopic radical prostatectomy: The Montsouris experience. J. Urol..

[10] Abrams P (2002). Standardisation sub-committee of the international continence society. The standardisation of terminology of lower urinary tract function: Report from the Standardisation Sub-committee of the International Continence Society. Neurourol. Urodyn..

[11] Kojima Y (2013). Urinary incontinence after robot-assisted radical prostatectomy: Pathophysiology and intraoperative techniques to improve surgical outcome. Int. J. Urol..

[12] Lee J (2020). Retzius sparing robot-assisted radical prostatectomy conveys early regain of continence over conventional robot-assisted radical prostatectomy: A propensity score matched analysis of 1863 patients. J. Urol..

[13] Dalela D (2017). A pragmatic randomized controlled trial examining the impact of the Retzius-sparing approach on early urinary continence recovery after robot-assisted radical prostatectomy. Eur. Urol..

[14] Egan J (2020). Retzius-sparing robot-assisted radical prostatectomy leads to durable improvement in urinary function and quality of life versus standard robot-assisted radical prostatectomy without compromise on oncologic efficacy: Single-surgeon series and step-by-step guide. Eur. Urol..

[15] Checcucci E (2020). Retzius-sparing robot-assisted radical prostatectomy vs the standard approach: A systematic review and analysis of comparative outcomes. BJU Int..

[16] Myers RP (2000). Puboperineales: Muscular boundaries of the male urogenital hiatus in 3D from magnetic resonance imaging. J. Urol..

[17] Matsubara A (2003). Topographic anatomy of the male perineal structures with special reference to perineal approaches for radical prostatectomy. Int. J. Urol..

[18] Soga H (2008). Topographical relationship between urethral rhabdosphincter and rectourethralis muscle: A better understanding of the apical dissection and the posterior stitches in radical prostatectomy. Int. J. Urol..

[19] Wilson PD (1983). Posterior pubo-urethral ligaments in normal and genuine stress incontinent women. J. Urol..

[20] Petros PE, Ulmsten U (1995). Urethral pressure increase on effort originates from within the urethra, and continence from musculovaginal closure. Neurourol. Urodyn..

[21] Petros PE, Ulmsten UI (1990). An integral theory of female urinary incontinence: Experimental and clinical considerations. Acta. Obstet. Gynecol. Scand. Suppl..

[22] Ulmsten U (1996). An ambulatory surgical procedure under local anesthesia for treatment of female urinary incontinence. Int. Urogynecol. J. Pelvic. Floor. Dysfunct..

[23] Viereck V (2006). Role of bladder neck mobility and urethral closure pressure in predicting outcome of tension-free vaginal tape (TVT) procedure. Ultrasound. Obstet. Gynecol..

[24] Sohn DW (2014). Pelvic floor musculature and bladder neck changes before and after continence recovery after radical prostatectomy in pelvic MRI. J. Magn. Reson. Imaging..

[25] Centemero A (2010). Preoperative pelvic floor muscle exercise for early continence after radical prostatectomy: A randomised controlled study. Eur. Urol..

[26] Milios JE, Ackland TR, Green DJ (2019). Pelvic floor muscle training in radical prostatectomy: A randomized controlled trial of the impacts on pelvic floor muscle function and urinary incontinence. BMC Urol..

[27] Chang JI (2016). Preoperative pelvic floor muscle exercise and postprostatectomy incontinence: A systematic review and meta-analysis. Eur. Urol..

[28] Student V (2017). Advanced reconstruction of vesicourethral support (ARVUS) during robot-assisted radical prostatectomy: One-year functional outcomes in a two-group randomised controlled trial. Eur. Urol..

[29] Kojima Y (2014). Bladder neck sling suspension during robot-assisted radical prostatectomy to improve early return of urinary continence: A comparative analysis. Urology.

[30] Cestari A (2017). Intraoperative retrograde perfusion sphincterometry to evaluate efficacy of autologous vas deferens 6-branch suburethral sling to properly restore sphincteric apparatus during robot-assisted radical prostatectomy. J. Endourol..

[31] Canvasser NE (2016). Posterior urethral suspension during robot-assisted radical prostatectomy improves early urinary control: A prospective cohort study. J. Endourol..

[32] Chen YC, Lin PH, Jou YY, Lin VC (2017). Surgical treatment for urinary incontinence after prostatectomy: A meta-analysis and systematic review. PLoS ONE.

